# Ambulatory Melanoma Care Patterns in the United States

**DOI:** 10.1155/2013/689261

**Published:** 2013-08-21

**Authors:** Andrew L. Ji, Michael R. Baze, Scott A. Davis, Steven R. Feldman, Alan B. Fleischer

**Affiliations:** ^1^Galderma Center for Dermatology Research, Department of Dermatology, Wake Forest School of Medicine, Winston-Salem, NC 27157-1071, USA; ^2^Nova Southeastern University/Broward Health Medical Center, Department of Dermatology, Fort Lauderdale, FL 33315, USA; ^3^Galderma Center for Dermatology Research, Department of Pathology, Wake Forest School of Medicine, Winston-Salem, NC 27157-1071, USA; ^4^Galderma Center for Dermatology Research, Department of Public Health Sciences, Wake Forest School of Medicine, Winston-Salem, NC 27157-1071, USA; ^5^Wake Forest University School of Medicine, Department of Dermatology, Medical Center Boulevard, Winston-Salem, NC 27157-1071, USA

## Abstract

*Objective*. To examine trends in melanoma visits in the ambulatory care setting. *Methods*. Data from the National Ambulatory Medical Care Survey (NAMCS) from 1979 to 2010 were used to analyze melanoma visit characteristics including number of visits, age and gender of patients, and physician specialty. These data were compared to US Census population estimates during the same time period. *Results*. The overall rate of melanoma visits increased (*P* < 0.0001) at an apparently higher rate than the increase in population over this time. The age of patients with melanoma visits increased at approximately double the rate (0.47 year per interval year, *P* < 0.0001) of the population increase in age (0.23 year per interval year). There was a nonsignificant (*P* = 0.19) decline in the proportion of female patients seen over the study interval. Lastly, ambulatory care has shifted towards dermatologists and other specialties managing melanoma patients and away from family/internal medicine physicians and general/plastic surgeons. *Conclusions*. The number and age of melanoma visits has increased over time with respect to the overall population, mirroring the increase in melanoma incidence over the past three decades. These trends highlight the need for further studies regarding melanoma management efficiency.

## 1. Introduction

In 2013, the American Cancer Society estimates that there will be 76,690 new cases of melanoma diagnosed, with 61,300 being melanoma *in situ* [1]. Melanoma management costs the US billions of dollars in direct annual expenses [2]. Worldwide, during the past several decades, there has been a substantial increase in the incidence of melanoma, particularly among white populations [3, 4]. In the US, the incidence of melanoma also continues to rise. From 1973 to 1994, US melanoma incidence rates increased 154.4% in males (from 6.8 to 17.3 per 100,000) and 90.2% in females (from 6.1 to 11.6 per 100,000) [5]. The melanoma incidence rates during 2006 to 2010 reveal a similar trend in males (27.4 per 100,000) and females (16.7 per 100,000), nearly quadrupling in men and tripling in women over the past three to four decades [6]. More specifically, during this period, the incidence rates per 100,000 persons were 31.9 and 20.0 in white men and women, respectively; 4.7 and 4.4 in Hispanic men and women, respectively; 1.6 and 1.1 in Asian men and women, respectively; and 1.1 and 1.0 in black men and woman, respectively [6].

According to the National Cancer Institute Surveillance Epidemiology End Results data, from 2006 to 2010, the median age at diagnosis for melanoma was 61 years, with approximately 65% of cases occurring in those 55 years and older [6]. Current estimates of lifetime risk for Americans experiencing melanoma are 1 in 37 for men and 1 in 56 for women, which contrasts with the lifetime risk of 1 in 1,500 for Americans born in 1935 [7, 8]. With increasing incidence, an understanding of the ambulatory management patterns of melanoma care has national significance. We sought to characterize how office visits for melanoma are changing.

## 2. Methods

The number of melanoma visits in physician office based settings was estimated using data from the National Ambulatory Medical Care Survey (NAMCS). The NAMCS is conducted by the Division of Health Care Statistics of the National Center for Health Statistics to provide data on a representative sample of ambulatory physician office visits in USA. The complex sampling frame examines nonhospital-based physician office visits, and the primary unit of analysis is the visit. These data are weighted to produce national estimates that describe the utilization of ambulatory medical care services in USA. The number of visits was obtained by identifying melanoma visits (ICD-9-CM diagnosis code of 172.0 to 172.9) for all patients for the interval years 1979 to 2010. Tumor staging information is not included in diagnosis coding. A total of 872,197 records estimating the experience of 26 billion office visits were searched to identify 819 unique melanoma visit records which estimate the experience of 17.4 million visits to USA physicians. Data extracted contain information about patient demographics and specialty of treating physicians. All analysis was performed using the survey procedures (SURVEYFREQ, SURVEYREG, and SURVEYMEANS) contained within SAS 9.1.3 (Cary, NC, USA).

Population data were derived from the US Census Bureau (http://www.census.gov/) over the representative period using both historical data (http://www.census.gov/popest/data/historical/index.html) and current estimate data (http://www.census.gov/popest/data/index.html).

## 3. Results

**Figure 1:**
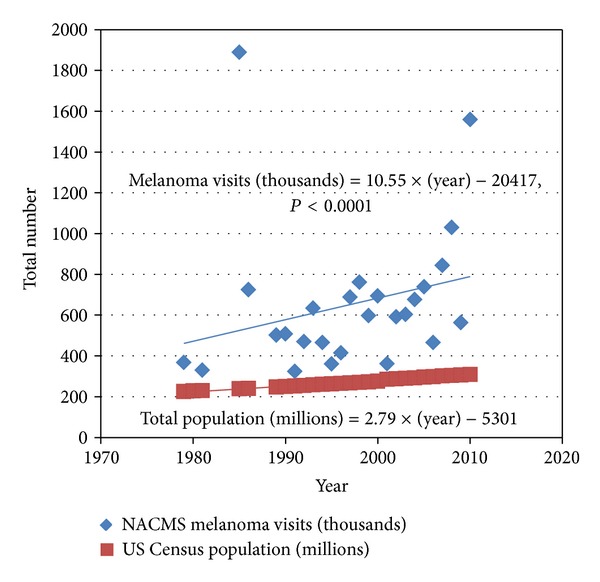
Estimated number of melanoma visits (thousands) from the US National Ambulatory Medical Care Survey (NACMS) from 1979 to 2010 compared to total US population (millions).

To test the hypothesis that ambulatory melanoma visit rates have changed in relation to the population, we compared NAMCS melanoma visits to US Census population estimates. Estimates of the numbers of melanoma visits demonstrated a significant increase in the interval from 1979 to 2010 (*P* < 0.0001). These data, as well as US population data from the Census Bureau, are plotted in Figure 1. The rate of increase in number of melanoma visits appears to be higher than the rate of increase in US population. Because the estimates are derived using widely disparate methods, direct comparison of the two datasets cannot be performed.

**Figure 2:**
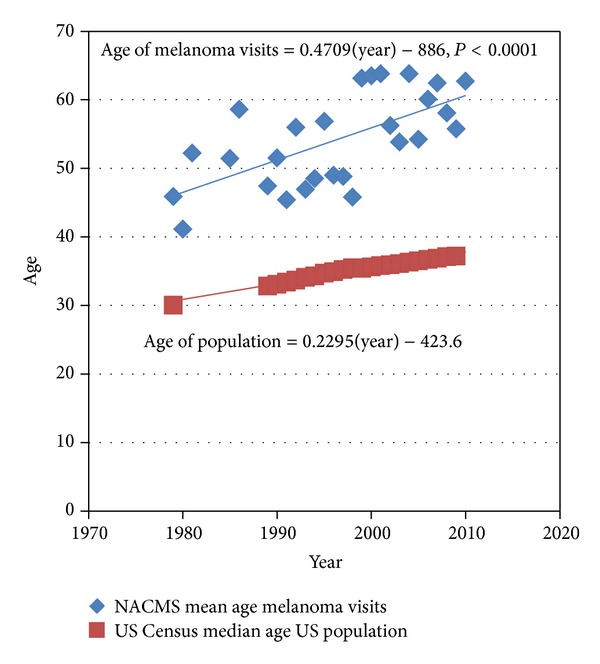
Mean age of melanoma visits from the US National Ambulatory Medical Care Survey (NAMCS) from 1979 to 2010 compared to median age of US population.

The age of patients with melanoma visits appears to be rising at approximately double the rate of the rise in the population increase in age (Figure 2). We estimated that the age of patients with melanoma visits increased by 0.47 year per interval year from 1979 to 2010, whereas the aging rate of the population was 0.23 year per interval year for the general population. Note that population means were obtained for the NAMCS melanoma visits, whereas only population medians from the census data were available.

**Figure 3:**
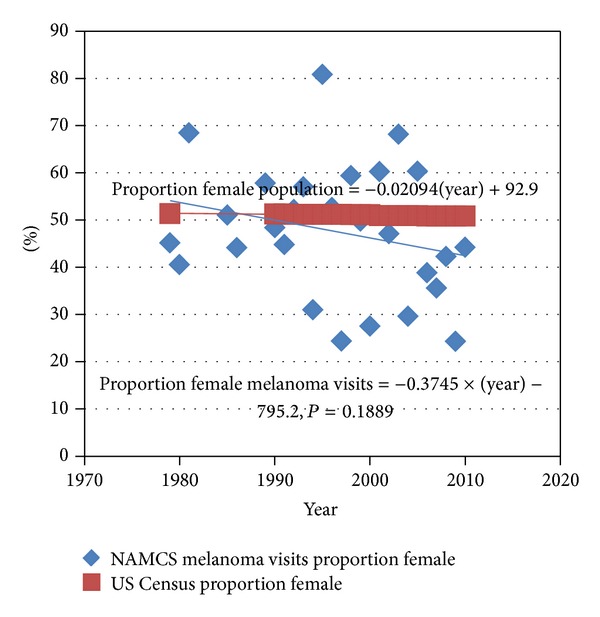
Proportion of female melanoma visits from the US National Ambulatory Medical Care Survey (NAMCS) from 1979 to 2010 compared to proportion female in population.

To test the hypothesis that the gender distribution of melanoma visits has changed over time, Figure 3 shows the proportion of the visits by female patients over the study interval, which demonstrates a nonsignificant (*P* = 0.19) decline in the proportion of female patients seen over the study interval.

**Figure 4:**
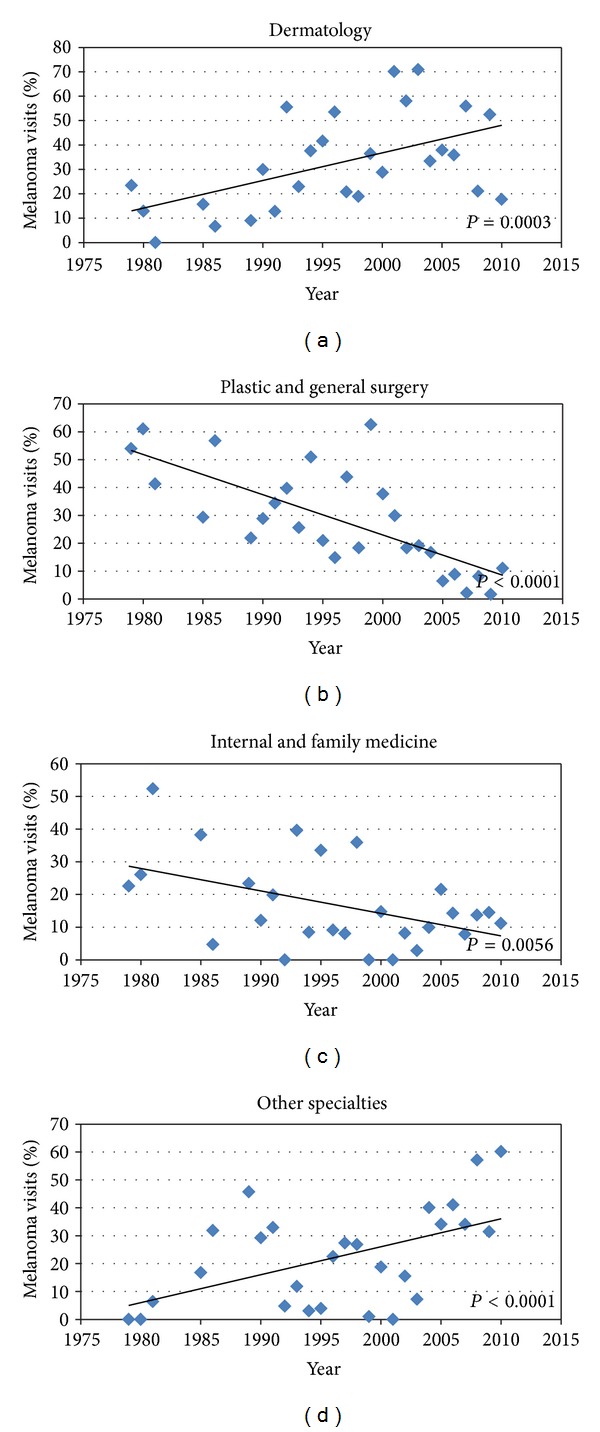
Proportion of melanoma visits by specialty from the US National Ambulatory National Care Survey (NAMCS) from 1979 to 2010 in (a) dermatology, (b) plastic and general surgery, (c) internal and family medicine, and (d) other specialties.

Ambulatory care by different specialties appears to be changing over time, with a significant increase in the proportion of visits to dermatologists (*P* = 0.0003) and a corresponding decrease in the proportion of visits to family/internal medicine physicians (*P* = 0.0056) and general/plastic surgeons (*P* < 0.0001) (Figures 4(a), 4(b), and 4(c)). There was also an increasing trend in melanoma management by all other specialties (*P* < 0.0001) (Figure 4(d)).

## 4. Discussion

Our findings show that the overall rate of melanoma visits has increase in comparison to the baseline increase in population (Figure 1). Given that the melanoma visits sampled in this study could be either initial visits or follow-up visits for a previously diagnosed melanoma, we are unable to comment on melanoma incidence from these data. However, other studies indicate that the incidence of melanoma has been on the rise over the past several decades [5, 6], which would help explain why the number of visits has significantly increased. The increase in number of visits also suggests that management in the ambulatory care setting has accommodated the rising number of melanoma patients. There are a few explanations for this trend. Partly contributing to the increasing melanoma incidence are better detection practices and earlier detection of melanoma [9], which both occur most often in the ambulatory care setting. It has also been shown that the rise in incidence is mainly attributable to thin melanomas, while the number of intermediate or thick melanomas has remained stable [10, 11]. Recent data show that dermatologists are diagnosing more melanomas and are doing so at earlier stages than any other specialty [12, 13]. They are now managing cases previously referred to surgical specialties. With dermatologists increasingly handling more diagnosis responsibilities, these factors could easily contribute to the observed increase in number of visits in the ambulatory care setting. It would be important to discern whether ambulatory care visits by these patients are the most efficient way to manage their disease. Efficiency in this context could be described as the least number of patient visits necessary in the appropriate management of a given melanoma.

Our study also reveals that the age of patients with melanoma visits increasing at approximately twice the rate of the population age increase (Figure 2). The mean age of patients with melanoma visits was about 45 years in 1979 and about 60 years in 2010. This suggests that the age at which people are diagnosed with melanoma is advancing over time. An analysis of the crude and birth-cohort adjusted age-specific rates of melanoma suggests that rates will continue to increase as earlier cohorts age [14]. However, it seems likely that this trend is due to more than just an aging population; otherwise the mean age of melanoma visits would track more closely with the mean age of the population. A reasonable explanation could include the changing solar exposure practices over the past decades and the long latency to the development of melanoma. If preventative practices were being appropriately employed, we would expect to see fewer young people developing melanoma, thereby contributing to the higher age at diagnosis. In recent years, however, adolescents and young adults have been reported to be at increased risk of skin cancer due to suboptimal sunscreen use, high rates of sunburning, and tanning bed use [15]. Time will tell if these practices will lead to a higher incidence of melanoma in the younger populations. Further concerning evidence is that in US females, melanoma is reported to be the most common cancer in the 25 to 29 age group, and the second most common cancer in women aged 30 to 34 years [16]. As mentioned previously, better detection practices over the years could also explain an older age at diagnosis given the long latency period of the disease, especially if a diagnosis was missed at an earlier age.

During the time period examined by our study, the proportion of melanoma visits by women appears to closely correlate with the proportion of females in the population (Figure 3). However, albeit small, there is still a decline in the proportion of melanoma visits by females. This contrasts the fairly constant proportion of females in the population. In a study of melanoma incidence and mortality in US whites from 1969 to 1999, Geller et al. demonstrated increased melanoma incidence in men and women (aged 20 to 65 years or older), with greater increases noted in the older male population [17]. Other studies support these findings [18, 19]. With evidence demonstrating a greater incidence in males, we would expect to see a greater proportion of male melanoma visits. Possibly explaining this trend is that men may be less likely to visit a physician or maintain compliance with follow-up visits. Studies have demonstrated that while women have higher medical care service utilization and are more likely to use outpatient medical services, men are less likely to have physician office visits or preventative care visits [20–22]. Further, men are presenting to the physician with thicker melanomas than women, possibly necessitating in-patient treatment, which is not accounted for by the NAMCS data [23]. Women living longer and having lower melanoma mortality can also influence this trend. While men and women aged 20 to 44 had a melanoma mortality decrease of 29% and 39%, respectively, men 65 years and older had a 157% mortality increase [17]. This was a 3-fold greater increase than for women of the same age.

In an effort to contain healthcare expenditures, greater emphasis has been placed on primary care physicians (PCPs) providing healthcare, as specialists, including dermatologists, are assumed to be more costly. In terms of management of skin conditions, this positions PCPs in a difficult situation, as much of their practice involves management of chronic conditions like hypertension and diabetes mellitus, and relatively little time diagnosing and treating skin disease. Fleischer Jr. et al. found that some of the most common cutaneous diagnoses made by family physicians were diagnosed at least 10 times more frequently by dermatologists [12]. Smith et al. demonstrated the large disparity in experience between dermatologists and other physicians with outpatient management of skin cancer [24]. The authors found that the majority of skin cancer visits (81%) were managed by dermatologists, with the remaining visits being managed by nondermatologists. Consistent with this, our data shows that over a few decades, dermatologists received more melanoma visits, while melanoma visits to family and internal medicine physicians have significantly decreased over this time period (Figures 4(a)–4(c)). Given that the majority of melanoma visits have been going to dermatologists, nondermatologists may have relatively little clinical exposure to melanoma. Studies show that most PCPs referred patients presenting with suspicious appearing pigmented lesions to a dermatologist, as many PCPs did not feel confident in their ability to recognize melanoma and thought their training was not adequate to prepare them to diagnose and manage pigmented lesions [25, 26]. In surveys of US medical students and residents, the majority reported little to no training in skin care examinations and felt a lack of competency and confidence in performing these [27–29]. With a declining proportion of melanoma visits going to PCPs, this lack of confidence may only worsen. Conversely, for dermatologists, the increase in melanoma clinical experience has likely led to an increased level of confidence and expertise in melanoma management. The effect of greater physician experience on improved quality of care and medical cost reduction has been demonstrated [30, 31].

The rise in melanoma visits to dermatologists over the past three decades has also corresponded with a decline in visits to surgeons (Figure 4), a finding that is also reflected in other studies. In a study looking at the surgical management of melanoma by dermatologists and surgeons, the percentage of the total patients treated by dermatologists rose from 18% in 1979–84 to 57% in 1991–97, while those treated by general surgeons decreased from 58% to 15% and from 23% to 13% for plastic surgeons over the same period [32]. Many factors could contribute to this trend, including public recognition of dermatologists as skin care experts, and dermatology residencies providing more surgical and procedural training than in the past, allowing graduates to offer more comprehensive skin care than before [33].

As NAMCS data is derived from office-based physician practices in USA, one of the limitations of this study is the lack of data from hospital inpatient and military medical facilities. In the case of advanced melanomas managed by the general or plastic surgeons, this could underestimate both the overall total number of melanoma visits and the number of melanoma visits seen by these providers. With the NAMCS database limited to physicians primarily involved in outpatient care outside the federal system, this could lead to potential selection bias or sampling error. Another limitation of this study is that the melanoma visits could represent either new diagnosis or followups on previously diagnosed melanoma. To better estimate the new cases of melanoma, perhaps visits associated with excisions would provide a more accurate assessment. Lastly, the NAMCS data reflect the ability of the various practitioners to make an accurate diagnosis. For those with less experience in dealing with pigmented lesions, misdiagnosis could occur. For example, it is not uncommon for a seborrheic keratosis to masquerade as melanoma, which could lead to an overestimation of melanoma visits. Likewise, if a practitioner is using a particular system for diagnosis, such as the Asymmetry, Borders, Color, and Diameter (ABCD) system, they potentially could misdiagnose a melanoma that did not fit this system. This could lead to either an underestimation or overestimation of melanoma visits.

## 5. Conclusion

The trends demonstrated in our study offer insight into characteristics of ambulatory melanoma visits over three decades. Specifically, our investigation revealed that both the number and age of melanoma visits has risen over time, mirroring the increase in incidence of melanoma. Further, no gender differences were identified over time. Primary care physicians and surgeons have decreased their contribution to care, while dermatologists and other specialties have increased their contribution of care. While these findings allow for the possible exploration and understanding of contributing factors, they also highlight the need for further studies. For example, a study looking at the number of visits associated with a given type and stage of melanoma could provide information that would allow for a more accurate assessment of melanoma management efficiency. Not only would this have the potential of raising the standard of care but also lower healthcare expenditures. With dermatologists expanding their scope of practice to include more surgical and procedural dermatology, along with the general recognition of dermatologists as the skin cancer experts, the disparities in melanoma visits between dermatologists and nondermatologists will likely continue to grow.

## References

[1] American Cancer Society (2013). *Cancer Facts and Figures 2013*.

[2] Bickers DR, Lim HW, Margolis D (2006). The burden of skin diseases: 2004. *Journal of the American Academy of Dermatology*.

[3] Diepgen TL, Mahler V (2002). The epidemiology of skin cancer. *British Journal of Dermatology*.

[4] Schaffer JV, Rigel DS, Kopf AW, Bolognia JL (2004). Cutaneous melanoma—past, present, and future. *Journal of the American Academy of Dermatology*.

[5] Hall HI, Miller DR, Rogers JD, Bewerse B (1999). Update on the incidence and mortality from melanoma in the United States. *Journal of the American Academy of Dermatology*.

[6] Howlader N, Noone AM, Krapcho M *SEER Cancer Statistics Review, 1975–2010*.

[7] Jemal A, Siegel R, Xu J, Ward E (2010). Cancer statistics. *CA: A Cancer Journal for Clinicians*.

[8] Rigel DS, Carucci JA (2000). Malignant melanoma: prevention, early detection, and treatment in the 21st century. *Ca: A Cancer Journal for Clinicians*.

[9] Lee KC, Weinstock MA (2009). Melanoma is up: are we up to this challenge?. *Journal of Investigative Dermatology*.

[10] Lipsker DM, Hedelin G, Heid E, Grosshans EM, Cribier BJ (1999). Striking increase of thin melanomas contrasts with stable incidence of thick melanomas. *Archives of Dermatology*.

[11] Demierre M, Chung C, Miller DR, Geller AC (2005). Early detection of thick melanomas in the United States: beware of the nodular subtype. *Archives of Dermatology*.

[12] Fleischer AB, Herbert C, Feldman SR, O’Brien F (2000). Diagnosis of skin disease by nondermatologists. *American Journal of Managed Care*.

[13] Chen SC, Pennie ML, Kolm P (2006). Diagnosing and managing cutaneous pigmented lesions: primary care physicians versus dermatologists. *Journal of General Internal Medicine*.

[14] Dennis LK (1999). Melanoma incidence by body site: effects of birth-cohort adjustment. *Archives of Dermatology*.

[15] Geller AC, Colditz G, Oliveria S (2002). Use of sunscreen, sunburning rates, and tanning bed use among more than 10,000 US children and adolescents. *Pediatrics*.

[16] Brochez L, Naeyaert J-M (2000). Understanding the trends in melanoma incidence and mortality: where do we stand?. *European Journal of Dermatology*.

[17] Geller AC, Miller DR, Annas GD, Demierre M, Gilchrest BA, Koh HK (2002). Melanoma incidence and mortality among US whites, 1969–1999. *Journal of the American Medical Association*.

[18] Desmond RA, Soong S (2003). Epidemiology of malignant melanoma. *Surgical Clinics of North America*.

[19] Jemal A, Devesa SS, Hartge P, Tucker MA (2001). Recent trends in cutaneous melanoma incidence among whites in the United States. *Journal of the National Cancer Institute*.

[20] Pinkhasov RM, Wong J, Kashanian J (2010). Are men shortchanged on health? Perspective on health care utilization and health risk behavior in men and women in the United States. *International Journal of Clinical Practice*.

[21] Bertakis KD, Azari R, Helms LJ, Callahan EJ, Robbins JA (2000). Gender differences in the utilization of health care services. *Journal of Family Practice*.

[22] Cleary PD, Mechanic D, Greenley JR (1982). Sex differences in medical care utilization: an empirical investigation. *Journal of Health and Social Behavior*.

[23] Schwartz JL, Wang TS, Hamilton TA, Lowe L, Sondak VK, Johnson TM (2002). Thin primary cutaneous melanomas: associated detection patterns, lesion characteristics, and patient characteristics. *Cancer*.

[24] Smith ES, Feldman SR, Fleischer AB, Leshin B, McMichael A (1998). Characteristics of office-based visits for skin cancer: dermatologists have more experience than other physicians in managing malignant and premalignant skin conditions. *Dermatologic Surgery*.

[25] Friedman KP, Whitaker-Worth DL, Grin C, Grant-Kels JM (2004). Melanoma screening behavior among primary care physicians. *Cutis*.

[26] Kirsner RS, Muhkerjee S, Federman DG (1999). Skin cancer screening in primary care: prevalence and barriers. *Journal of the American Academy of Dermatology*.

[27] Lee M, Hodgson CS, Wilkerson L (2002). Predictors of self-perceived competency in cancer screening examinations. *Journal of Cancer Education*.

[28] Moore MM, Geller AC, Zhang Z (2006). Skin cancer examination teaching in US medical education. *Archives of Dermatology*.

[29] Wise E, Singh D, Moore M (2009). Rates of skin cancer screening and prevention counseling by US medical residents. *Archives of Dermatology*.

[30] Chang W, Li T, Lin C (2003). The effect of physician experience on costs and clinical outcomes of laparoscopic-assisted vaginal hysterectomy: a multivariate analysis. *Journal of the American Association of Gynecologic Laparoscopists*.

[31] Sosa JA, Bowman HM, Tielsch JM, Powe NR, Gordon TA, Udelsman R (1998). The importance of surgeon experience for clinical and economic outcomes from thyroidectomy. *Annals of Surgery*.

[32] Mckenna DB, Marioni JC, Lee RJ, Prescott RJ, Doherty VR (2004). A comparison of dermatologists', surgeons' and general practitioners' surgical management of cutaneous melanoma. *British Journal of Dermatology*.

[33] Todd MM, Miller JJ, Ammirati CT (2002). Dermatologic surgery training in residency. *Dermatologic Surgery*.

